# Supplementary material for: Time-lapsed imaging of nanocomposite scaffolds reveals increased bone formation in dynamic compression bioreactors
Source: Commun Biol. 2021 Jan 25;4:110. doi: 10.1038/s42003-020-01635-4 (PMC7835377; doi:10.1038/s42003-020-01635-4)
Supplement: Supplementary file 2 — Supplementary Information [file 42003_2020_1635_MOESM2_ESM.pdf]

SUPPLEMENTARY INFORMATION FOR MANUSCRIPT

Time-lapsed imaging of nanocomposite scaffolds reveals increased bone formation in dynamic compression bioreactors

*Gian Nutal Schädli,[1,2] Jolanda R. Vetsch[1], Robert P. Baumann[2], Anke M. de Leeuw[1], Esther Wehrle[1], Marina Rubert[1] and Ralph Müller[1]**

[1]Institute for Biomechanics, Department of Health Sciences and Technology, ETH Zurich, Zurich, Switzerland
*Corresponding author: ram@ethz.ch

[2]Particle Technology Laboratory, Department of Mechanical and Process Engineering, ETH Zurich, Zurich, Switzerland

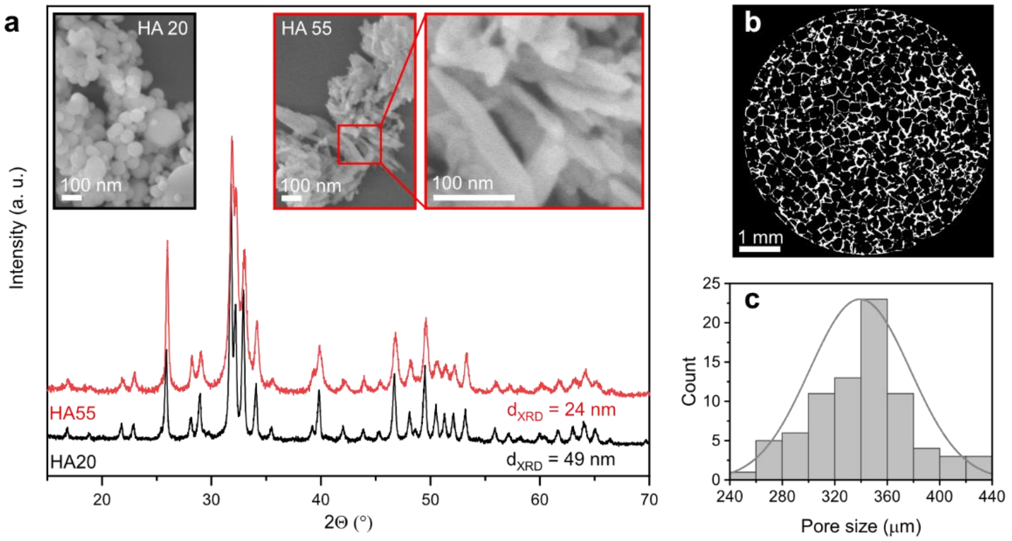

Fig. S1: Nanoparticle and bone scaffold characterization. a, XRD pattern of spherical HA20 and needle-like HA55 particles. The insets show corresponding SEM images. **b**, Micro-CT image of a cross-sectional slice of a representative HA55-PLGA (u) scaffold after thresholding into a binary image. **c,** Pore size distribution of that scaffold.

1

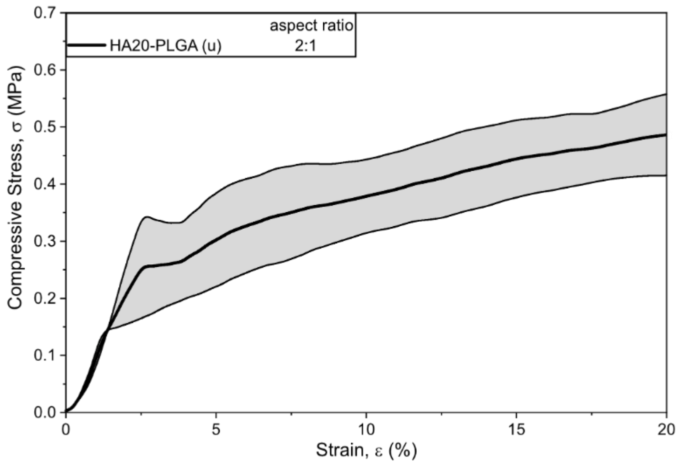

Fig. S2: Stiffness of nanoparticle reinforced polymer nanocomposite bone scaffolds. Compressive stress-strain measurement of HA20PLGA (u) scaffolds with a 2:1 aspect ratio (n = 5), prepared by SCPL modified with ultrasonication of nanofiller and pressure molding, denoted by (u). The bold line represents the mean and the shaded area the s.d.

2

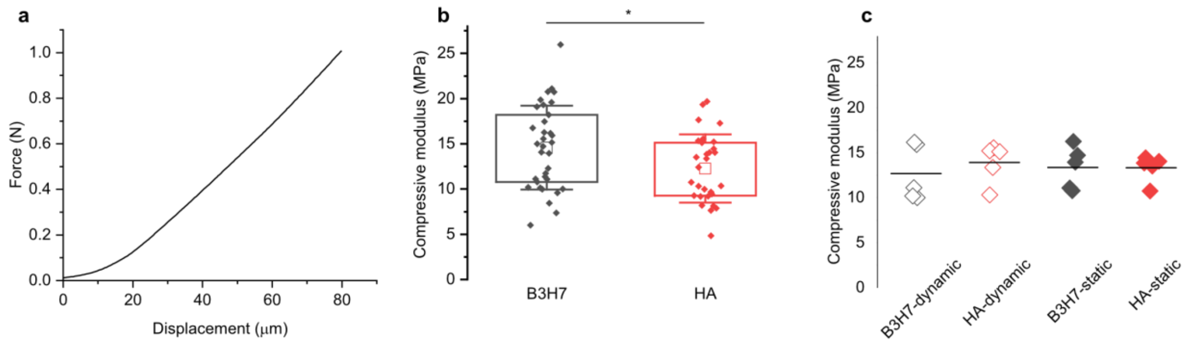

Fig. S3: Scaffold screening and selection for bioreactor culture. a, Nondestructive compressive stress-strain measurement of a representative HA scaffold. **b**, Compressive modulus of all prepared B3H7 (n = 34) and HA (n = 29) scaffolds. The compressive modulus was obtained from linear fits in the region from 3-5 N. The box plots represent the mean with 1st and 3rd quartile. The error bars represent the s.d. *P < 0.05, t-test. **c**, Compressive modulus of selected scaffolds that form the various groups for the bioreactor culture. The data is represented by the mean (n = 5) and individual values. There is no significant difference between the groups; one-way analysis of variance (ANOVA) and Bonferroni post-hoc tests. Shapiro-Wilk and Levene's Test were not significant.

Supplementary Note 1 - Loading control

To rule out that compressing the scaffolds would cause the shift, we first fully soaked a HA scaffold by immersion in Dulbecco's Modified Eagle Medium (DMEM) under vacuum. A first micro-CT scan and a histogram of the image proved that the scaffold was fully soaked with DMEM. Then, we applied 3 consecutive cyclic loadings and took again a micro-CT scan. In total, this procedure was repeated 5 times to simulate 5 weeks of normal culture while at the same time avoiding any mineral precipitation. The scaffold height dropped from the first to the last scan by 6.4%. Figure S4 shows that the probability density showed no shift but only a widening of the distribution. The observed changes are within the standard deviation of the distributions shown in Fig. 3h-i.

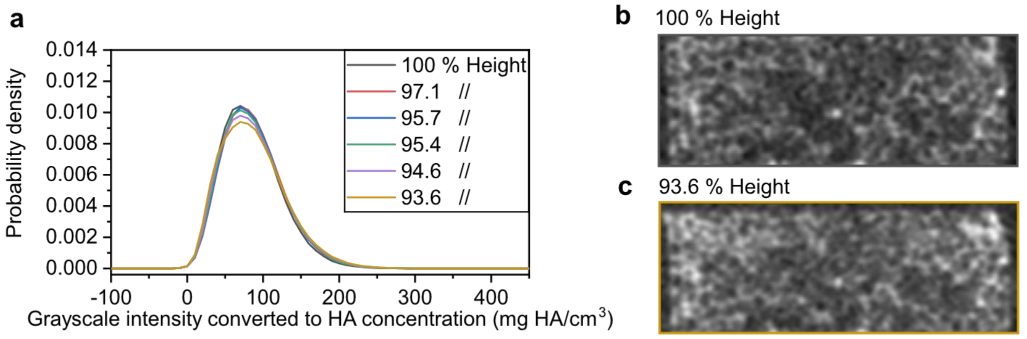

Fig. S4: Probability density distributions of compressed scaffolds. **a,** the change of the probability distribution of a scaffold that was compressed by applying repeatedly 3 consecutive loadings with 5

Hz, 3% strain using F_{thres} = 0.2 N. **b,** vertical micro-CT cross section of the scaffold at 100% height and **c,** after the last loading at 93.6% height.

Supplementary Note 2 - Time-lapsed micro-CT imaging and longitudinal monitoring, 5 Hz, 3% strain

The scaffold height loss, which was induced by cyclic compression, is barely visible and no obvious difference is detectable by eye between the scaffold cultured under static and dynamic condition (Figure S5a-d). Consistent with Fig. 3h-i, the histograms exhibit a shift in the probability density distributions between the two time-points. Note, that the scaffolds cultured under dynamic conditions (Fig. S5i), have the same height loss as the scaffold in the control experiment (Fig. S4). Nevertheless, they exhibit an obvious increase of the probability density at HA concentrations above the threshold, corroborating the fact that the observed changes in the probability density were not due to height loss. The distribution of the histogram is slightly more skewed towards higher densities (Fig. S5e-f vs. Fig. 3h-i), which can be attributed to the lower current intensity (165 vs. 177 µA) that had to be chosen due to technical issues with the X-Ray tube of the micro-CT. Consistent with Fig. 3, more mineral matured in scaffolds cultured under dynamic conditions compared to static condition. However, because of the skewed histogram, a difference in BV (10-50% at Week 6) between dynamic and static conditions was observed for scaffold mineral densities > 175 mg HA/cm^3 (Fig. S5g-h). Also, the total BV was higher in scaffolds cultured under dynamic conditions.

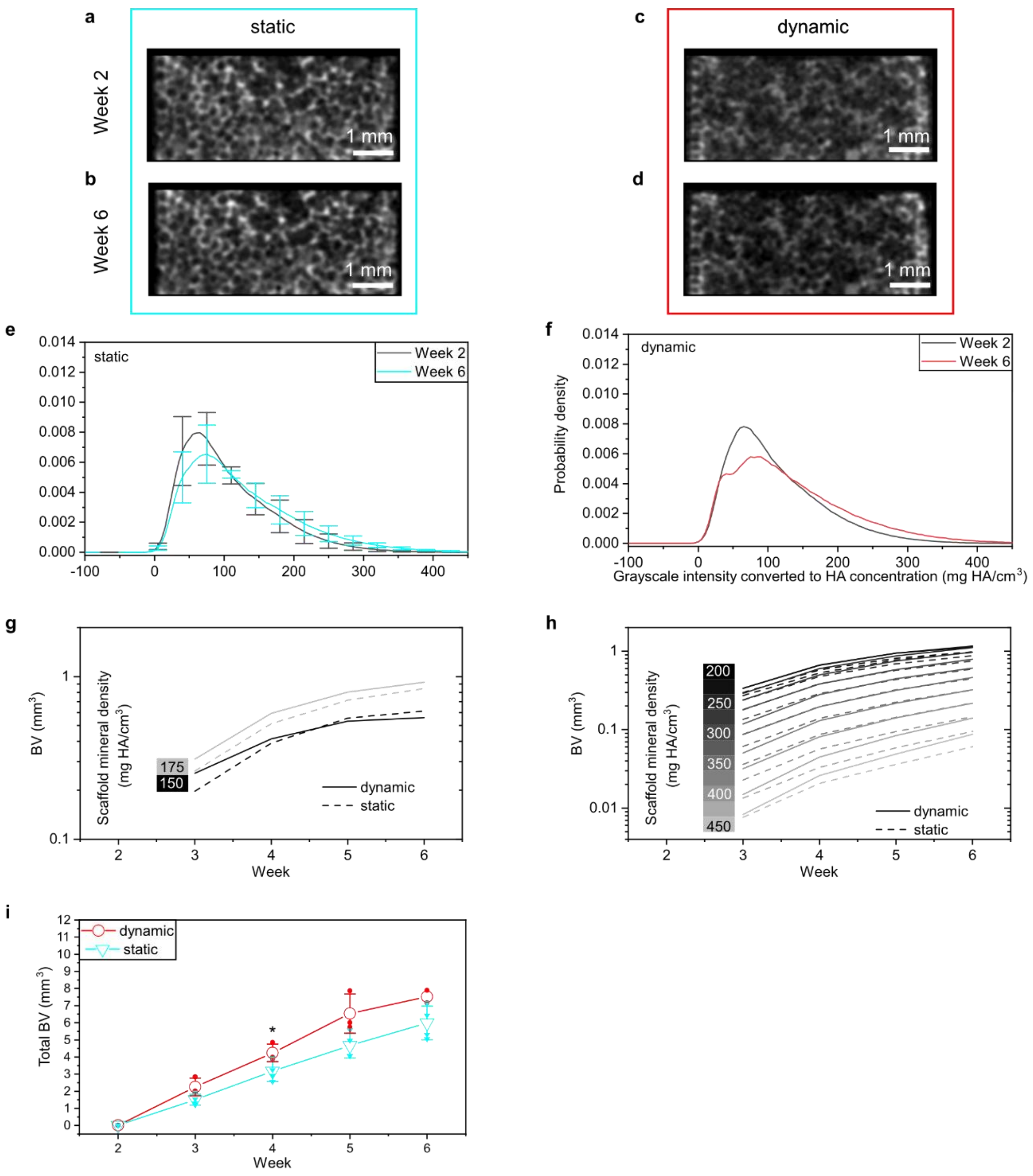

Fig. S5: Time-lapsed micro-CT monitoring of HA55-PLGA (u) scaffolds, 5 Hz and 3% strain. a, Micro-CT cross-sectional slice of a scaffold cultured under static condition from Week 2 and **b** Week 6, respectively of a scaffold cultured under dynamic condition (**c-d**). Histogram of such images from static (**e**, n = 4) and dynamic (**f**, n = 2) cultured scaffolds. Data is shown as mean ± s.d. **g,** BV for scaffold mineral density (SMD) 150 and 175 mg HA/cm^3, respectively **h** for SMD bins 200-450. The bin width is 25 mg HA/cm^3. **i,** Total BV as function of time for static (n = 4) and dynamic (n = 3 for Week 2-5, n = 2 for Week 6) culture conditions. Symbols represent the mean and error bars the s.d. (*P < 0.05); t-tests were used to highlight significant differences between both conditions for each time-point. Shapiro-Wilk and Levene's Test were not significant.

Table S1: Coefficient of variation (CV) for quiescent volume. Scaff. 1-7 correspond to the scaffolds in Fig. 4b.

CV (%)	Scaff. 1	Scaff. 2	Scaff. 3	Scaff. 4	Scaff. 5	Scaff. 6	Scaff. 7
top	2.19	1.38	2.40	2.24	0.89	0.94	1.58
bottom	2.16	1.08	1.41	2.00	1.8	1.1	0.28

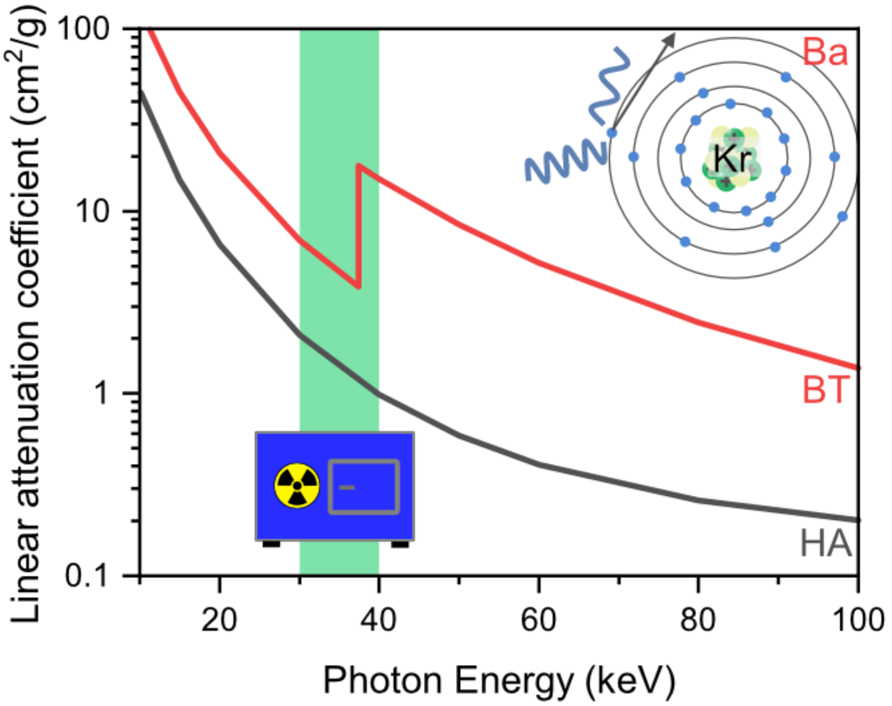

Fig. S6: Linear attenuation coefficients for Barium Titanate (BT) and Hydroxyapatite (HA). Values were calculated using NIST tables[1]. The green region indicates the photon energy corresponding to the micro-CT settings (45 kVp, 177 uA) used in this study. Insert shows electron shell model of Barium.

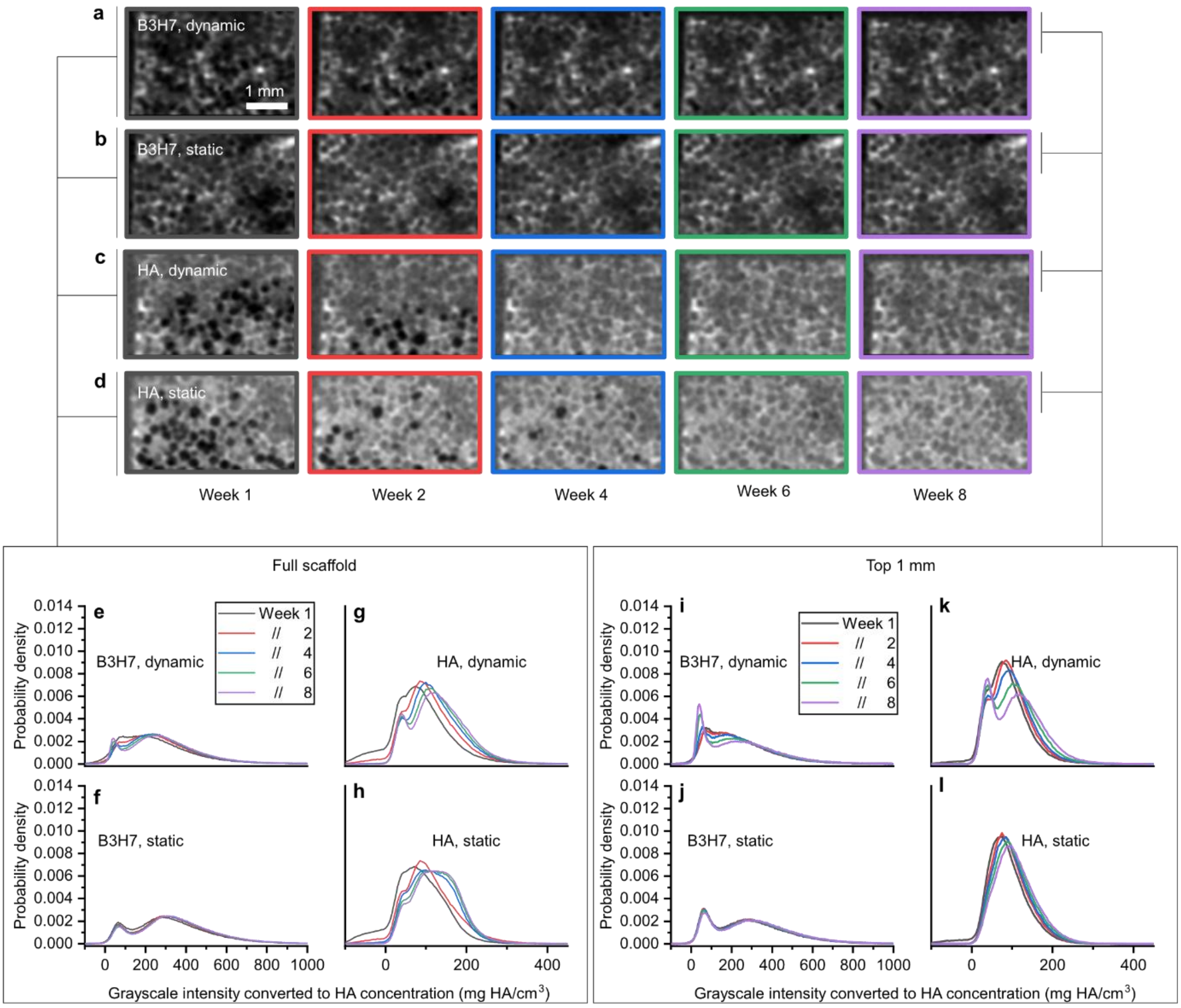

Figure S7: Longitudinal histograms for B3H7 and HA nanocomposite scaffolds. a-d, vertical cross sections from Week 1, 2, 4, 6 and 8 of a B3H7 (**a,b**) and HA (**c,d**) scaffold cultured dynamic (**a,c**) or static (**b,d**) conditions. **e-h**, histograms of 3D grayscale images of the full scaffold and **i-l** for the top 1 mm section. Air bubbles (black spots **a-d**, Week 1-4) were observed during the culture in the control medium and also confirmed by the corresponding histograms, displaying voxels with grayscale intensities corresponding to negative HA concentrations. No air bubbles were captured by the histogram confined to the top 1 mm for scans after week 2. Note, B3H7 scaffolds show voxel densities up to 900 mg/HA/cm^3 because of the larger attenuation coefficient of BT, hindering direct comparison to HA using absolute values of single scans.

Supplementary References

1. Hubbell, J. H. & Seltzer, S. M. Tables of X-Ray Mass Attenuation Coefficients and Mass Energy-Absorption Coefficients (version 1.4). National Institute of Standards and Technology (1995). https://dx.doi.org/10.18434/T4D01F.
