# Supplementary material for: Time-lapsed imaging of nanocomposite scaffolds reveals increased bone formation in dynamic compression bioreactors
Source: Commun Biol. 2021 Jan 25;4:110. doi: 10.1038/s42003-020-01635-4 (PMC7835377; doi:10.1038/s42003-020-01635-4)
Supplement: Supplementary file 3 — Description of Additional Supplementary Files [file 42003_2020_1635_MOESM3_ESM.pdf]

Description of Additional Supplementary Files

File: Supplementary Data 1

Description: Source data underlying the graphs in the paper.
