# Supplementary material for: Time-lapsed imaging of nanocomposite scaffolds reveals increased bone formation in dynamic compression bioreactors
Source: Commun Biol. 2021 Jan 25;4:110. doi: 10.1038/s42003-020-01635-4 (PMC7835377; doi:10.1038/s42003-020-01635-4)
Supplement: Supplementary file 5 — Reporting Summary [file 42003_2020_1635_MOESM5_ESM.pdf]

nature research

Corresponding author(s): Ralph Müller

Last updated by author(s): Dec 3, 2020

Reporting Summary

Nature Research wishes to improve the reproducibility of the work that we publish. This form provides structure for consistency and transparency in reporting. For further information on Nature Research policies, see our Editorial Policies and the Editorial Policy Checklist.

Statistics

For all statistical analyses, confirm that the following items are present in the figure legend, table legend, main text, or Methods section.

n/a	Confirmed	
☐	☒	The exact sample size (*n*) for each experimental group/condition, given as a discrete number and unit of measurement
☐	☒	A statement on whether measurements were taken from distinct samples or whether the same sample was measured repeatedly
☐	☒	The statistical test(s) used AND whether they are one- or two-sided<br>*Only common tests should be described solely by name; describe more complex techniques in the Methods section.*
☒	☐	A description of all covariates tested
☐	☒	A description of any assumptions or corrections, such as tests of normality and adjustment for multiple comparisons
☐	☒	A full description of the statistical parameters including central tendency (e.g. means) or other basic estimates (e.g. regression coefficient) AND variation (e.g. standard deviation) or associated estimates of uncertainty (e.g. confidence intervals)
☐	☒	For null hypothesis testing, the test statistic (e.g. *F*, *t*, *r*) with confidence intervals, effect sizes, degrees of freedom and *P* value noted<br>*Give P values as exact values whenever suitable.*
☒	☐	For Bayesian analysis, information on the choice of priors and Markov chain Monte Carlo settings
☒	☐	For hierarchical and complex designs, identification of the appropriate level for tests and full reporting of outcomes
☒	☐	Estimates of effect sizes (e.g. Cohen's *d*, Pearson's *r*), indicating how they were calculated

Our web collection on statistics for biologists contains articles on many of the points above.

Software and code

Policy information about availability of computer code

Data collection	Stress-strain: Instron MicroTester, 500 N load cell and standard desktop computer running Windows 7.<br>Non-destructive stress-strain: Zwick Z005 equipped with a 10 N load cell and standard desktop computer running Windows 7.<br>Force and displacement in bioreactor: LABVIEW National Instruments and standard desktop computer running Windows XP.<br>DNA content: Tecan Spark 10 and standard laptop running Windows 10.<br>Micro-CT: Scanco µCT 50 and 40 controlled by a computer cluster (HP Integrity rx2660, Intel Itanium)<br>Image acquisition: Hitachi S-4800, Slide Scanner Pannoramic 250, Leica TCS SP8 setup.
Data analysis	Mechanical Properties: Matlab R2019a<br>OriginPro 2019, Excel 2016, Fiji ImageJ 1.51p, BoneJ 1.4.2, Rstudio 1.1.456<br>Micro-CT image processing: IPL Scanco Medical AG Module 64-bit Version V5.42, Python 3.6.6, Scipy 1.2.0

For manuscripts utilizing custom algorithms or software that are central to the research but not yet described in published literature, software must be made available to editors and reviewers. We strongly encourage code deposition in a community repository (e.g. GitHub). See the Nature Research guidelines for submitting code & software for further information.

Data

Policy information about availability of data

All manuscripts must include a data availability statement. This statement should provide the following information, where applicable:

- Accession codes, unique identifiers, or web links for publicly available datasets
- A list of figures that have associated raw data
- A description of any restrictions on data availability

All the data that support the findings of this study are available from the corresponding author upon reasonable request.

Field-specific reporting

Please select the one below that is the best fit for your research. If you are not sure, read the appropriate sections before making your selection.

[x] Life sciences [ ] Behavioural & social sciences [ ] Ecological, evolutionary & environmental sciences

For a reference copy of the document with all sections, see nature.com/documents/nr-reporting-summary-flat.pdf

Life sciences study design

All studies must disclose on these points even when the disclosure is negative.

Sample size	No statistical analysis was performed to predetermine sample sizes. Sample sizes are similar to those generally employed in the field.
Data exclusions	Scaffolds cultured under dynamic conditions that were compressed > 5% strain due to malfunction of the mechanical stimulation unit were excluded (n = 2).
Replication	Cell culture experiments were conducted three times with at least 3 replicates. Static cell culture conditions were consistent. Dynamic conditions were conducted first with 1 Hz frequency, 5% strain and then with 5 Hz, 3% strain. Mechanical characterization were performed with 5 independent replicates from at least 2 batches.
Randomization	No randomization was required for experimental groups. The same FBS batch was used for all cell culture experiments.
Blinding	Blinding was not applicable to this study.

Reporting for specific materials, systems and methods

We require information from authors about some types of materials, experimental systems and methods used in many studies. Here, indicate whether each material, system or method listed is relevant to your study. If you are not sure if a list item applies to your research, read the appropriate section before selecting a response.

Materials & experimental systems

n/a	Involved in the study	
[ ]	[x]	Antibodies
[ ]	[x]	Eukaryotic cell lines
[x]	[ ]	Palaeontology and archaeology
[x]	[ ]	Animals and other organisms
[x]	[ ]	Human research participants
[x]	[ ]	Clinical data
[x]	[ ]	Dual use research of concern

Methods

n/a	Involved in the study	
[x]	[ ]	ChIP-seq
[x]	[ ]	Flow cytometry
[x]	[ ]	MRI-based neuroimaging

Antibodies

Antibodies used	Anti-osteocalcin: abcam ab93876, 1:200 dilution.<br>Secondary antibody donkey anti-rabbit IgG H&L Alexa Fluor-647: abcam ab150075, 1:1000 dilution.
Validation	The commercially available antibodies that were used in this study haven been cited multiple times in literature. Furthermore, following validation methods were conducted: 1) Secondary antibody was added alone without primary antibody addition, 2) No antibody was added, 3) the manufacturer has validated the antibody for use in the same species.

Eukaryotic cell lines

Policy information about cell lines

Cell line source(s)	The cell line was obtained from fresh human bone marrow (Lonza Walkersville, Inc).
Authentication	Cell surface antigens: CD14+, CD31−, CD34−, CD44+, CD71+ and CD105+
Mycoplasma contamination	The hMSCs were tested by a PCR mycoplasma-testing-kit (ATCC 30-1012K) as well as DNA Hoechst staining.
Commonly misidentified lines (See ICLAC register)	non available
